# Supplementary figures and images for: Combinations of DIPs and Dprs control organization of olfactory receptor neuron terminals in Drosophila
Source: PLoS Genet. 2018 Aug 13;14(8):e1007560. doi: 10.1371/journal.pgen.1007560 (PMC6107282; doi:10.1371/journal.pgen.1007560)

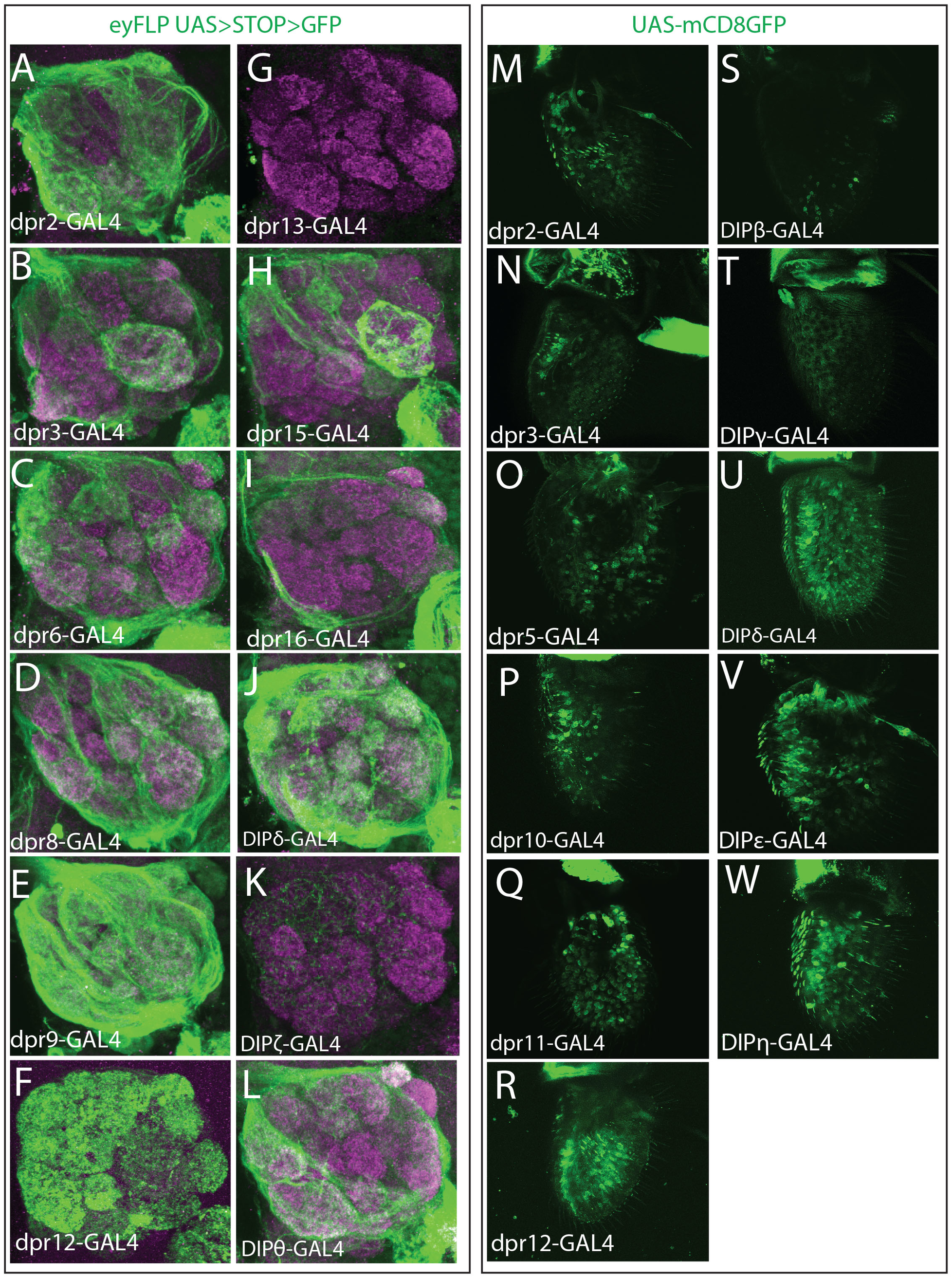

Supplement: S1 Fig — DIP and dpr-GAL4 reporters driving UAS>STOP>mCD8::GFP (green) not shown in Fig 2A–2L. DIP and dpr-GAL4s driving UAS-mCD8::GFP to visualize ORNs in the antenna (M-W). Most DIPs and dprs are expressed exclusively in ORNs although some are not expressed in the antenna at all. (TIF) [file pgen.1007560.s002.tif]

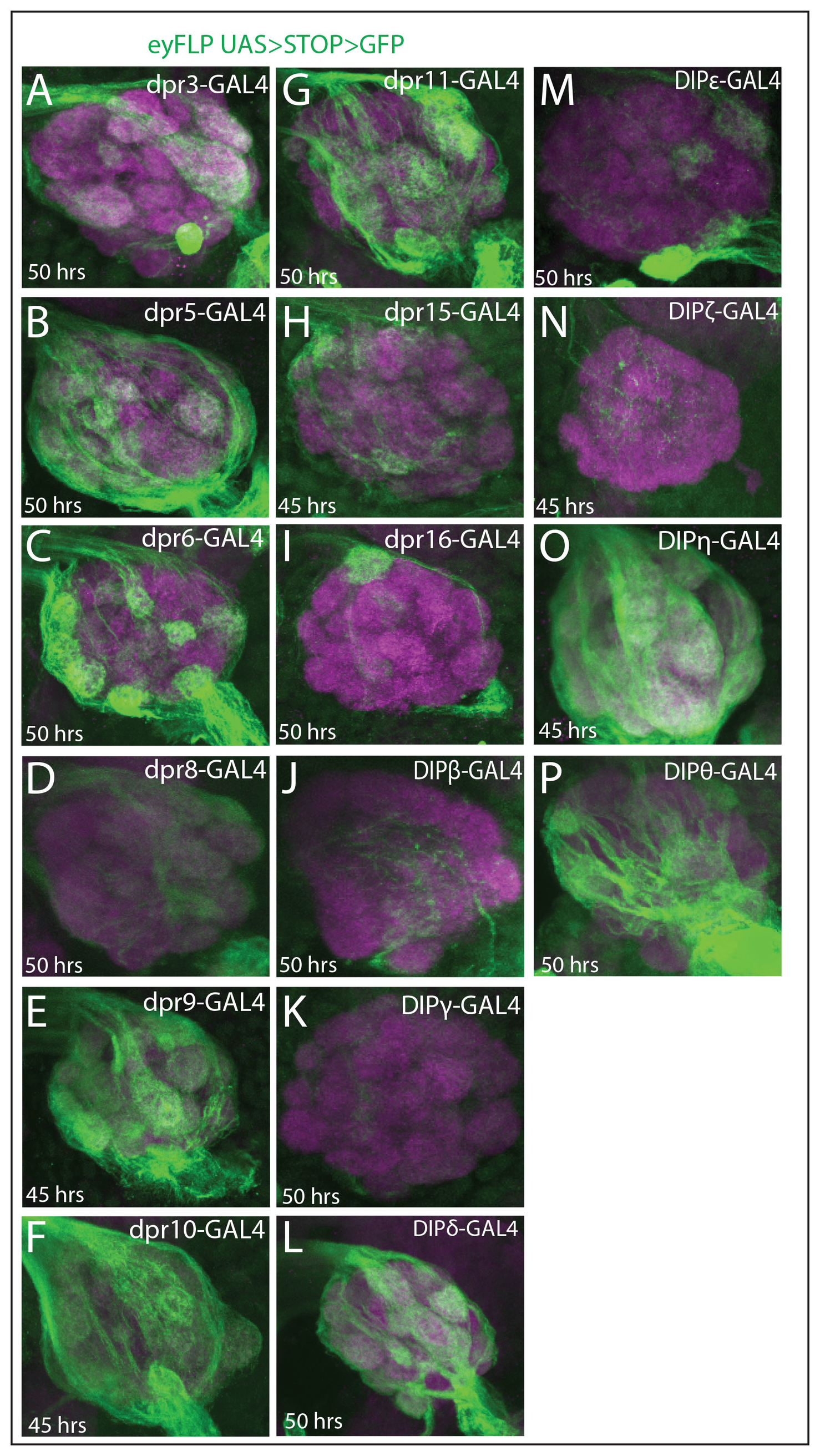

Supplement: S2 Fig — DIP/dpr-GAL4s driving the expression of UAS>STOP>mCD8::GFP (green) specifically in ORNs using ey driven flippase. Each DIP or dpr is expressed in a subset of ORN classes at this stage usually in fewer than in the adult. (TIF) [file pgen.1007560.s003.tif]

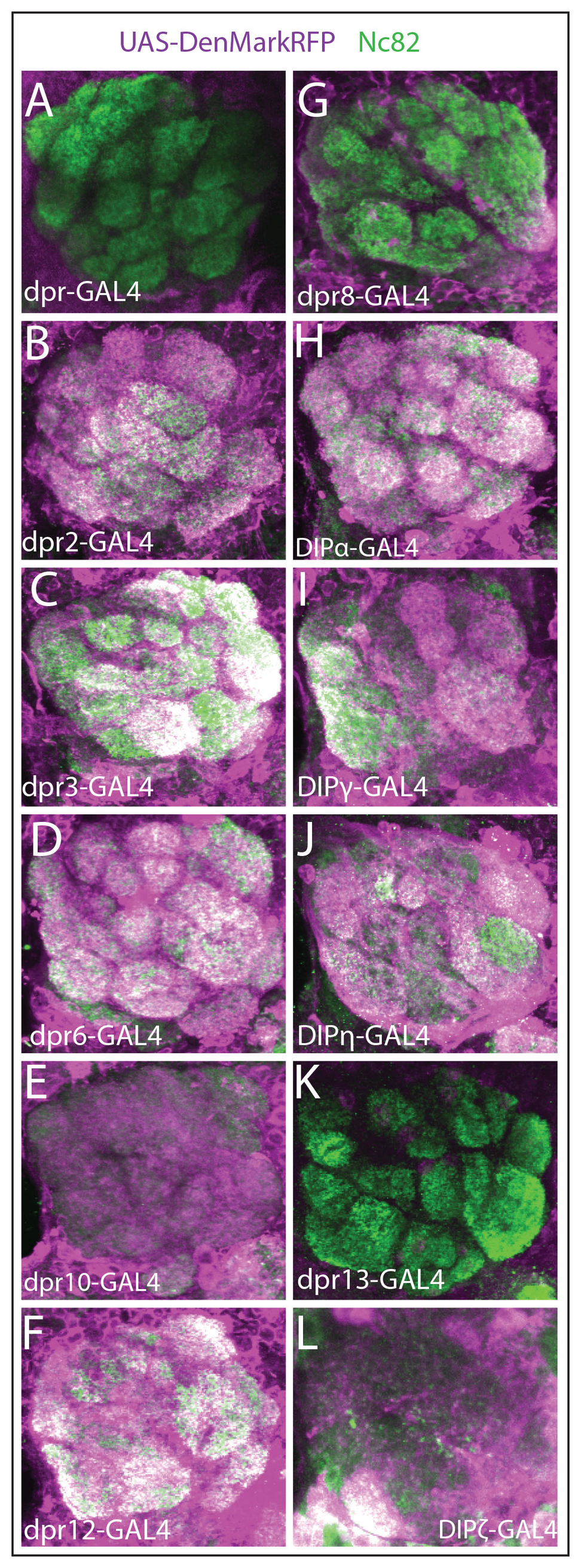

Supplement: S3 Fig — DIP and dpr-GAL4s were used to drive UAS-DenMark::RFP (magenta) with staining for neuropil in green (A-L). DIPs and dprs are expressed in subsets of projection neurons, all PNs or none. Binding partners make no discernable matching pattern between ORNs and PNs. (TIF) [file pgen.1007560.s004.tif]

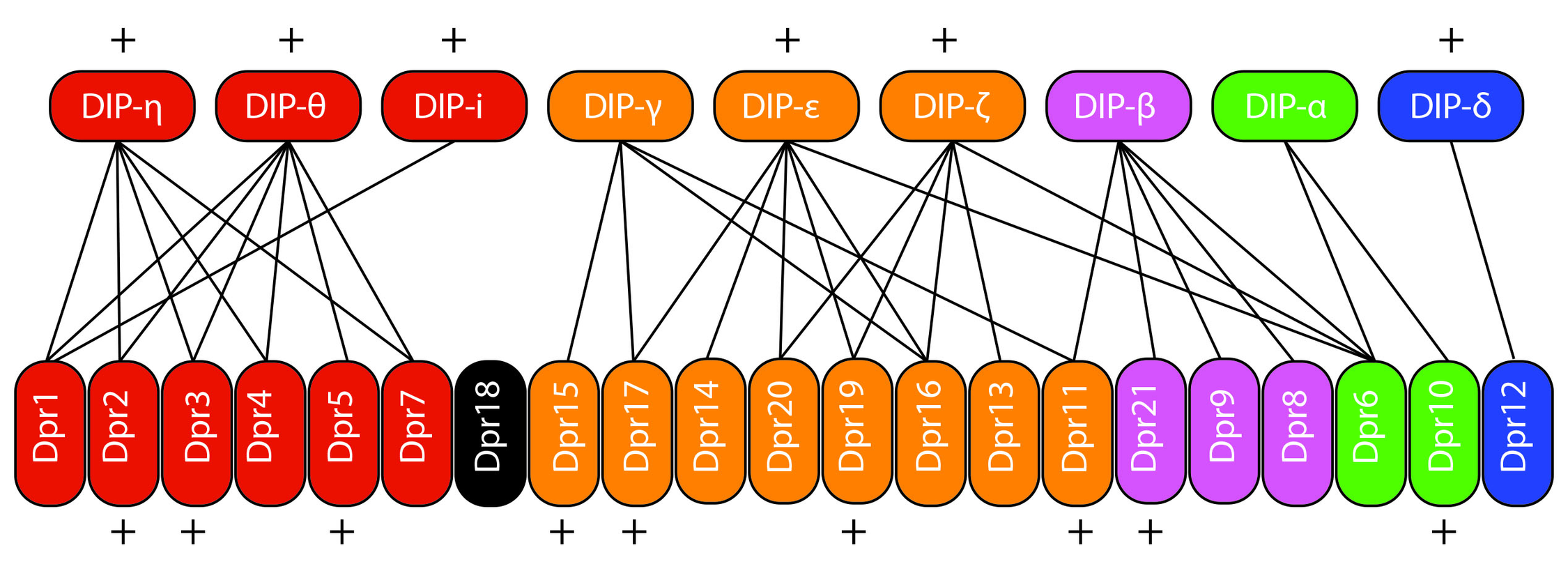

Supplement: S4 Fig — In addition, some DIPs and Dprs share homology with Kirrels. Approximately of half of DIP/Dpr proteins are homologous to mammalian Kirrels (+), which control glomerular formation in the mouse olfactory system. Homology is based upon FlyBase DIOPT scores. Adapted from [35,37]. (TIF) [file pgen.1007560.s005.tif]

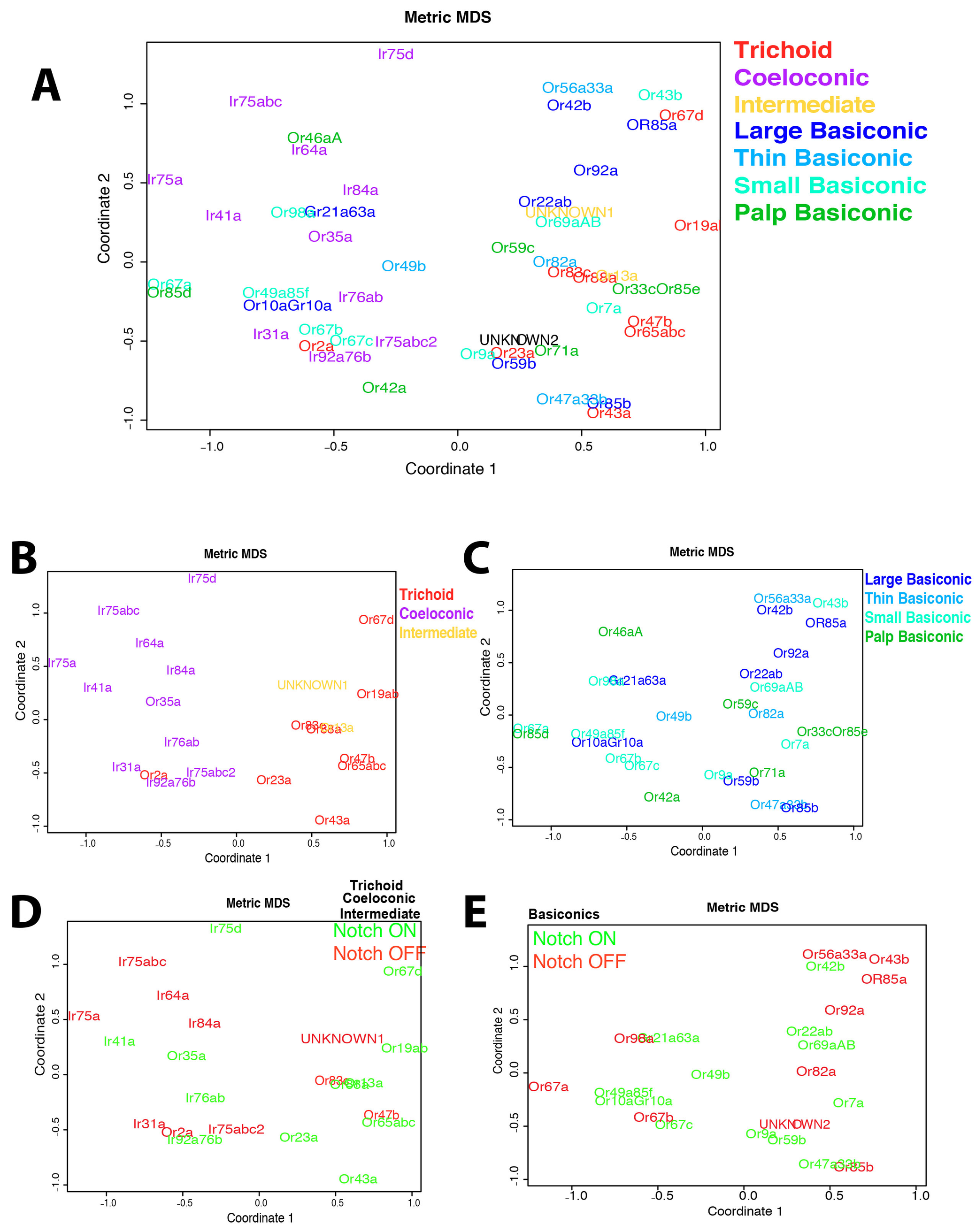

Supplement: S5 Fig — (A-C) ORN classes are labeled based upon sensilla morphological class (A), with coeloconics and trichoids (B) or basiconics (C) specifically. While no overall pattern is obvious, coeloconic classes and trichoid classes segregate away from each other. (D-E) ORN classes are colored based upon their Notch state, with coeloconics and trichoids (D) or basiconics (E) specifically. No pattern or segregation appears based upon Notch state. (TIF) [file pgen.1007560.s006.tif]

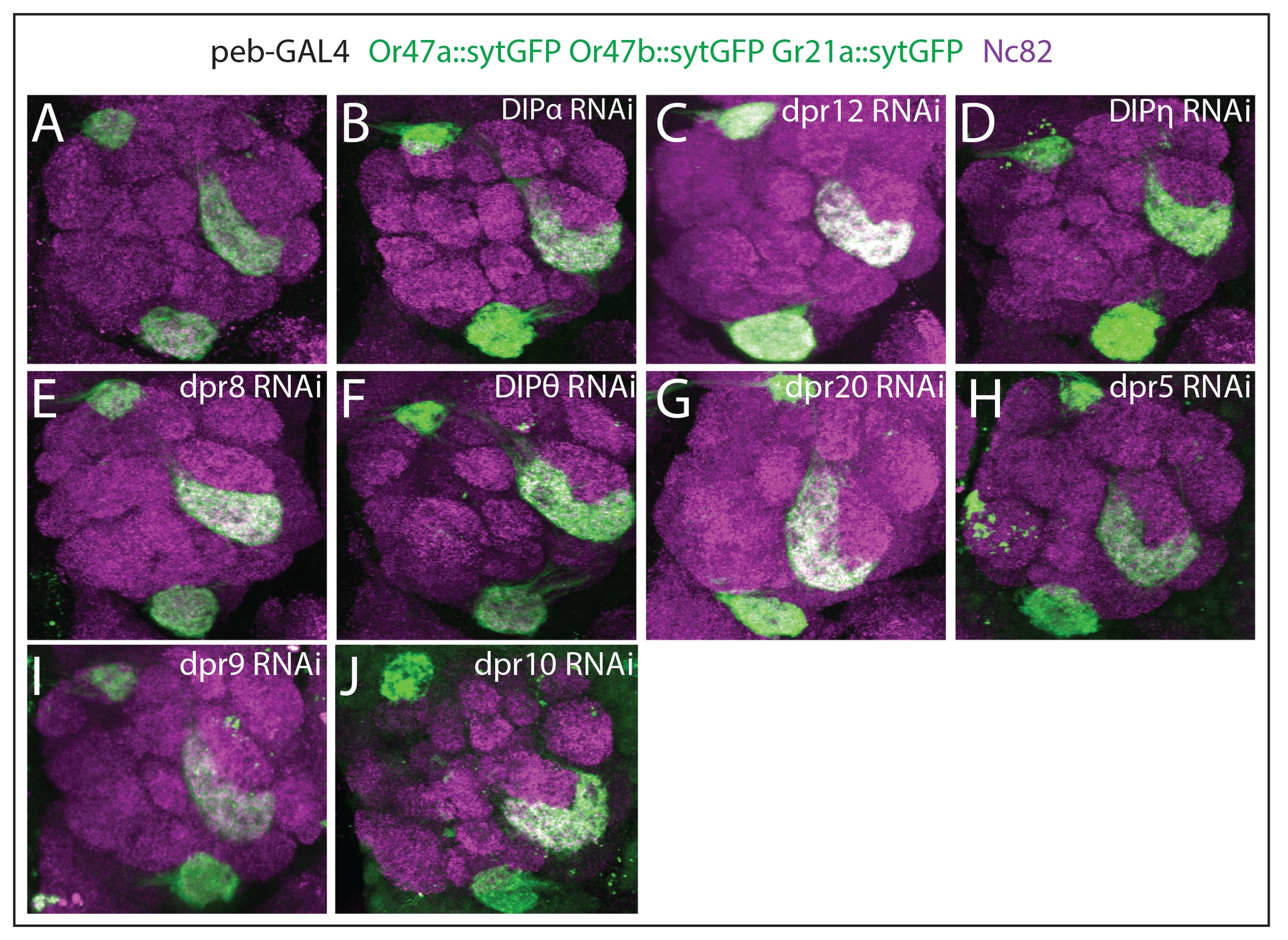

Supplement: S6 Fig — RNAi against single DIPs and dprs were driven in all ORNs with peb-GAL4 and axon terminals of Or47b, Or47a, and Gr21a were visualized using direct fusion reporters driving UAS-syt::GFP (A-J). No defects were detected in any of the conditions. (TIF) [file pgen.1007560.s007.tif]

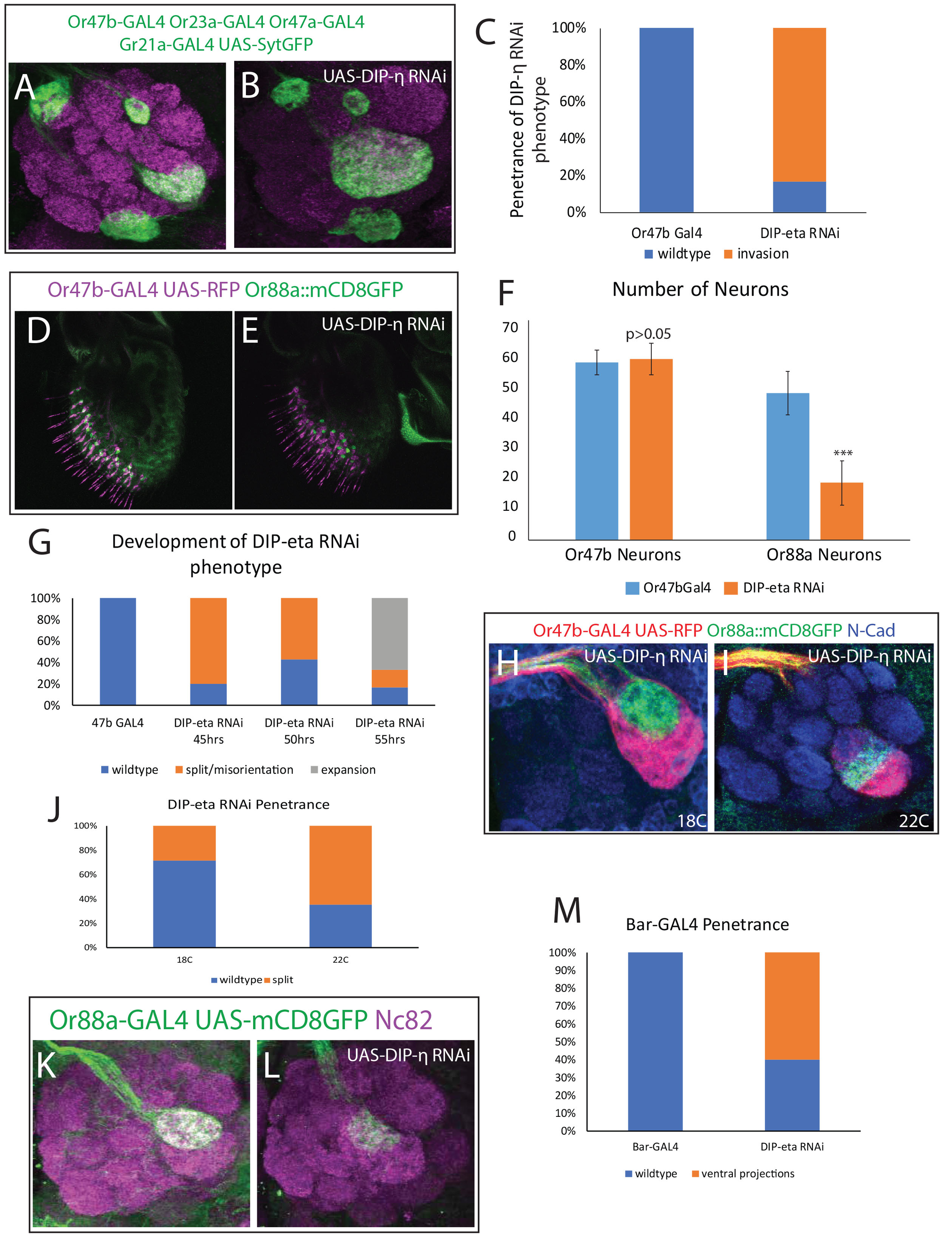

Supplement: S7 Fig — (A, B) Knock down of DIP-η with four OR-GAL4s specifically disrupts the morphology of the VA1v glomerulus, but not other classes of ORNs. (C) The expansion of the Or47b glomerulus is highly penetrant in DIP-η knock down, with 85% of individuals displaying invasion in one or both antennal lobes. (D-F) The number of Or47b ORNs (magenta) does not change with knock down of DIP-η (p>0.05), but the number of Or88a ORNs (green) is reduced by approximately half. This suggests that loss of DIP-η non-autonomously affects the generation or survival of Or88a ORNs in addition to glomerular morphology. (H, I) Knock down of DIP-η in flies raised at 22 and 18 °C. Flies raised at 18 °C display predominantly wildtype morphology of the VA1v (red) and VA1d (green) glomeruli. Flies raised at 22 °C displayed splitting of the VA1v glomerulus. (J) Spilt VA1v glomeruli were present in 68% of flies raised at 22 °C but only 29% of flies raised at 18 °C. (K, L) Knock down of DIP-η in Or88a ORNs using the Or88a-GAL4 driver does not disrupt the Or88a ORN target glomerulus. (M) Disruptions to the VA1d glomerulus were present in 60% of individuals in one or both antennal lobes when DIP-η was knocked down with the Bar-GAL4. (TIF) [file pgen.1007560.s008.tif]

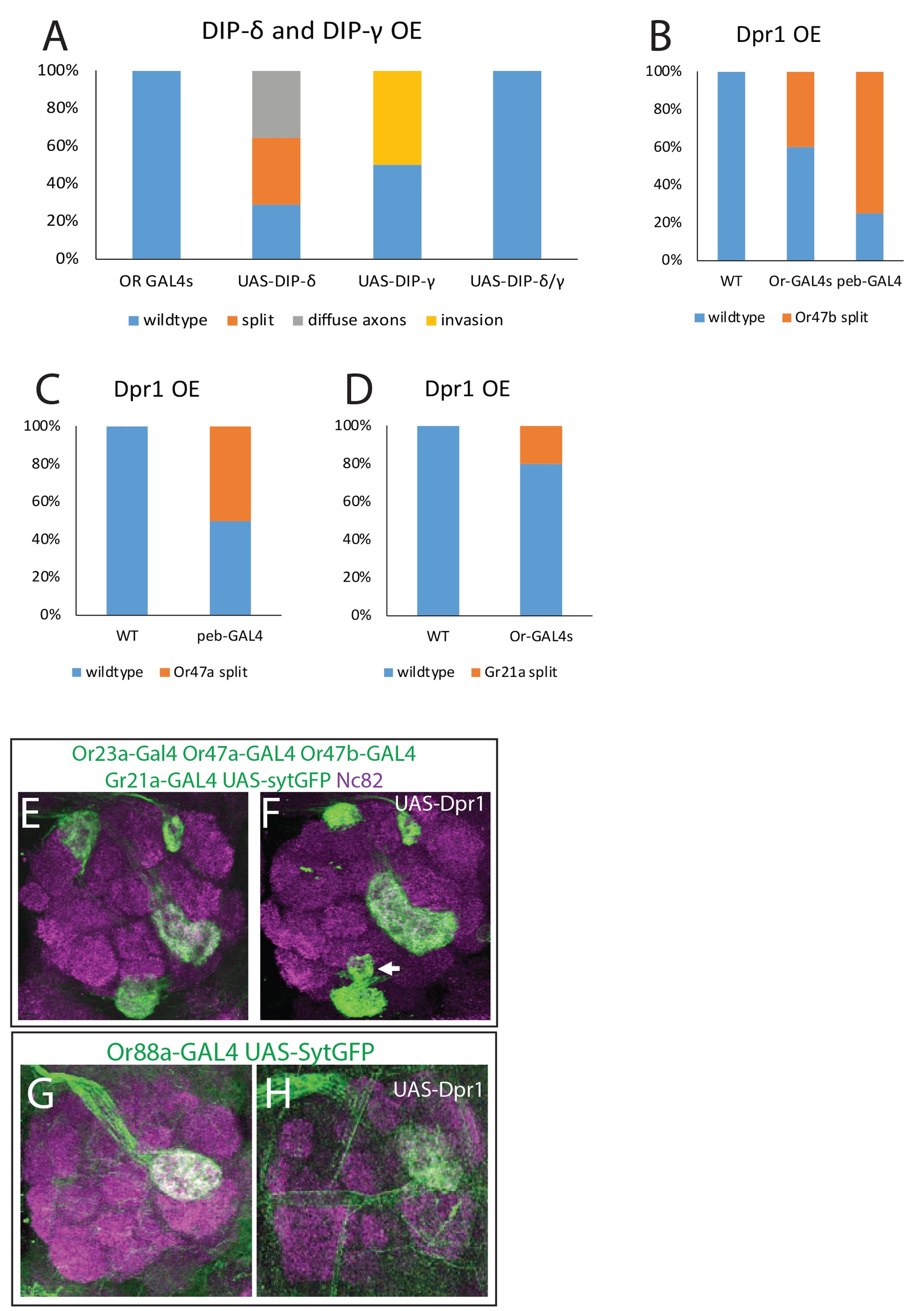

Supplement: S8 Fig — (A) 71% of individuals displayed either a split of the Or47b glomerulus or diffuse axons within the Or47b glomerulus during DIP-δ overexpression. 50% of individuals showed at least partial invasion of the Or88a glomerulus by Or47b axons when DIP-γ was expressed in Or47b neurons. All individuals displayed wildtype VA1v morphology when both genes were overexpressed. (B) 40% and 80% of individuals displayed splitting of the Or47b glomerulus when dpr1 was expressed in four ORN classes or all ORNs respectively. (C) Splitting of the Or47a glomerulus accompanied by the formation of an ectopic glomerulus was present in 50% of individuals when Dpr1 was expressed in all ORNs. (D). ~20% of individuals displayed a split of the V glomerulus during dpr1 overexpression. (E, F) Overexpression of dpr1 caused occasional splitting of the V glomerulus (F). (G, H) Overexpression of dpr1 in Or88a neurons did not disrupt the morphology of the VA1d glomerulus. (TIF) [file pgen.1007560.s009.tif]

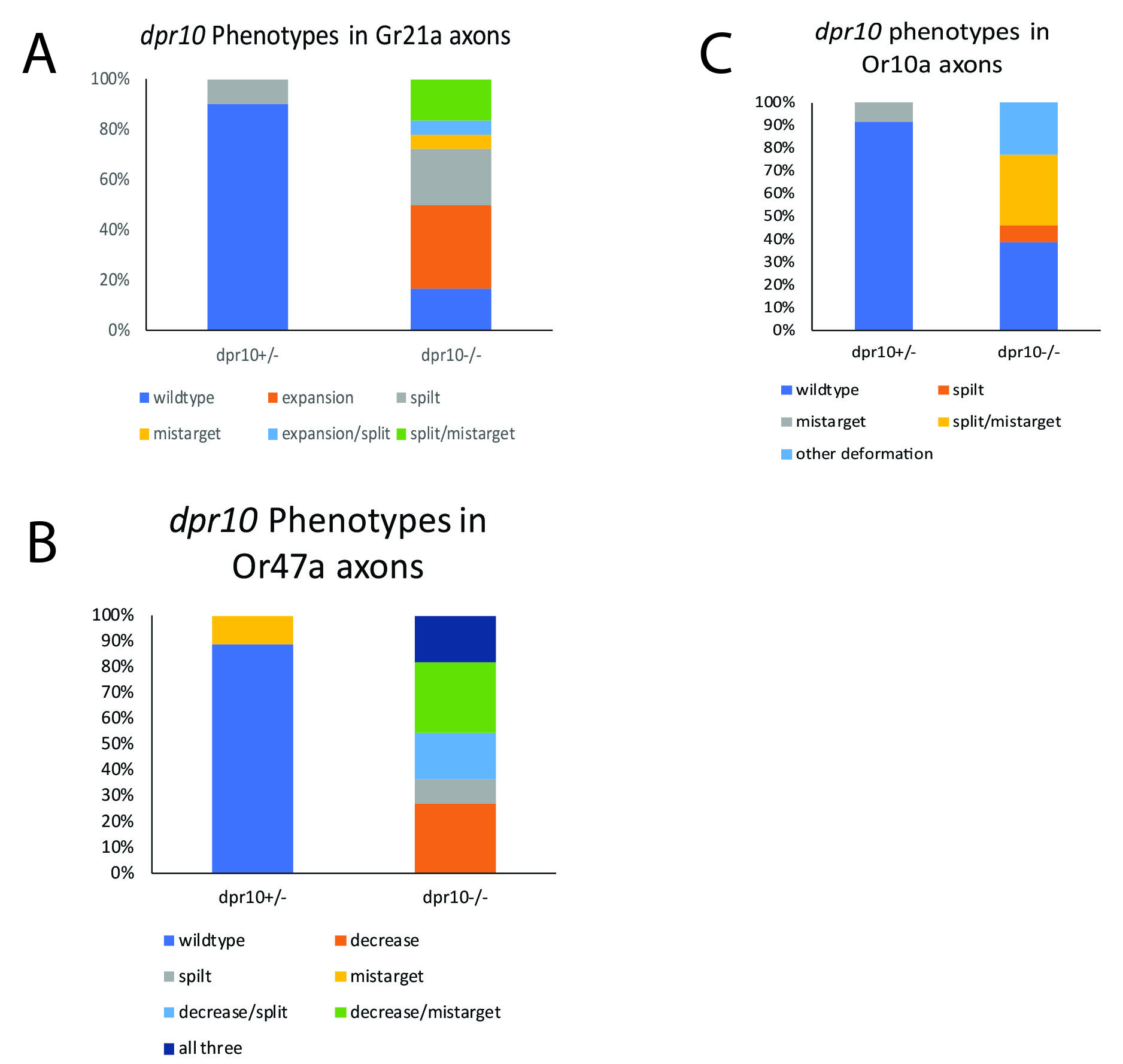

Supplement: S9 Fig — (A-C) Summary of penetrance and phenotypes observed in each class of neurons labeled. In each class, greater than 60% of individuals displayed a mutant phenotype in one or both antennal lobes. A minimum of 10 brains were imaged for each class of neurons. (TIF) [file pgen.1007560.s010.tif]
